# Supplementary material for: Are carbon emissions trading and green financial instruments synergistic? -Comprehensive quantitative research based on content analysis
Source: PLoS One. 2024 Mar 7;19(3):e0298601. doi: 10.1371/journal.pone.0298601 (PMC10919601; doi:10.1371/journal.pone.0298601)
Supplement: S1 Data — (ZIP) [file pone.0298601.s001.zip › Data/地级市Z_F工作报告.zip!/╣π╬≈/╙±┴╓╩╨/└┤╫╘╣1⁄2╓┌║┼í╛┬φ┐╦╩2╛▌═°í┐_2011.docx]

各位代表：

现在，我代表市人民政府，向大会作政府工作报告，请各位代表连同《玉林市国民经济和社会发展第十二个五年规划纲要（草案）》一并审议，并请市政协委员和列席会议的同志提出意见。

一、“十一五”时期的回顾和2010年工作

“十一五”时期，是我市经济社会快速发展、现代化建设阔步前进的重要时期。面对国内外环境的复杂变化和国际金融危机严重冲击的重大挑战，在自治区党委、政府和市委的正确领导下，在市人大、市政协的监督支持下，全市上下深入贯彻落实科学发展观，团结拼搏，开拓进取，胜利完成了“十一五”规划的主要目标任务。2010年与2005年相比，实现了“十三个翻番”，其中地区生产总值翻1.25番，人均生产总值翻1.18番，财政收入翻1.27番，工业增加值翻1.63番，服务业增加值翻1.16番，社会消费品零售总额翻1.24番，全社会固定资产投资翻2.23番，城镇居民人均可支配收入翻1.09番，农民人均纯收入翻1.04番，金融机构存款余额翻1.31番，金融机构贷款余额翻1.33番，实际利用外资翻1.96番，规模以上工业企业利润总额翻2.65番，开创了全市改革开放和现代化建设的崭新局面。

（一）经济持续较快增长，综合实力大幅提升。全市生产总值由2005年的352.6亿元增加到2010年的835.82亿元，年均增长14.4%，比“十五”时期快3.6个百分点；财政收入由28.64亿元增加到68.96亿元，年均增长19.2%，比“十五”时期快7.7个百分点；金融机构各项存款余额由310.07亿元增加到769.57亿元，年均增长19.9％。

（二）统筹城乡开局良好，城乡发展趋向协调。统筹城乡发展战略深入实施，玉东新区和7个县（市、区）的21个乡镇、村试点工作有效推进；城乡一体化基础设施建设大会战初见成效，市本级共建设项目91个，累计完成投资43亿元。农村基础设施建设力度加大，五年累计新（改）建农村公路3074公里，实现乡乡镇镇通硬化路目标，通硬化路行政村由42%提高到72%，农民出行条件明显改善。统筹城乡发展“四大基础工程”扎实推进，首批6个乡镇13个村的农村土地综合整治试点进展顺利。2010年，全市农民人均纯收入增速高于城镇居民人均可支配收入5.5个百分点。

（三）现代产业大步发展，经济结构明显优化。工业发展全面提速。2010年全市工业总产值首次突破1000亿元，达到1002.2亿元；工业增加值由2005年的104.41亿元增加到2010年的322.79亿元，年均增长19.9％；技改投资由20.88亿元增加到204.33亿元，年均增长57.8％。“强柴兴玉”战略成效明显，玉柴集团柴油机产销量连续10年位居国内同行首位，销售收入由2005年的121亿元增加到2010年的368.38亿元；规模以上工业企业由374家增加到741家，年销售收入超10亿元的强优企业从1家增加到7家；十大园区建设加快推进，五年累计新入园企业664家，全市入园企业达919家。实施上优汰劣，工业产业结构得到优化。机械、水泥陶瓷、健康、服装皮革、电子信息等五大产业集群总产值由2005年的128亿元增加到2010年的500.8亿元，对全市工业增长的贡献率达60%。全市工业化率从1.0提高到1.9。现代农业稳步发展。全市农林牧渔业总产值由2005年的166.5亿元增加到2010年的287.02亿元，年均增长6%。建设了12个部级万亩粮食高产示范区，粮食总产稳定增长，我市和北流、容县、陆川、博白、兴业等5个县（市）均获得全国粮食生产先进市、县称号；生猪和家禽养殖量、规模化养殖程度居广西各市之首；有7个产品获得中国地理标志产品保护，有2个产品获得中国名牌农产品、5个获得广西优质农产品称号。海峡两岸（广西玉林）农业合作试验区获国家批复成立，累计引进台湾农业新品种、新技术220项，建成生产示范基地、现代农业园55个。土地进一步向规模经营集中，全市土地流转面积达60.66万亩，占耕地总面积的22.86%。农业产业化水平领先全区，产业化经营组织由2005年的1992个增加到3747个，农业龙头企业由278家增加到560家，数量居全区首位。现代服务业快速增长。全市第三产业增加值由2005年的131.19亿元增加到2010年的292.47亿元，年均增长14.4%。建成玉林国际会展中心，银丰国际中药港、宏进农副产品批发市场等新一代市场竣工投入使用；五年共建设改造农家店1263家，配送中心16家；市场交易额由2005年的201亿元提高到2010年的456亿元，年均增长17.8%；全市公路货运周转量累计完成882.16亿吨公里，是“十五”时期的3.54倍；我市被商务部列为全国首批“流通领域现代物流示范城市”。金融、会展

、信息等服务业快速发展。五年累计接待游客2936.35万人次，是“十五”时期的1.76倍，全市旅游总收入由2005年的18.67亿元增加到2010年的50.54亿元，年均增长22%。经济结构持续优化。三次产业比重由2005年的28.5:34.3:37.2，优化提升到2010年的20.5：44.5：35。

（四）项目建设成效显著，发展动力不断增强。“十一五”是我市历史上建设重大项目数量最多、规模最大、完成投资最多的时期。全社会固定资产投资从2005年131.31亿元增加到2010年的615.56亿元，年均增长36.2%；五年累计完成投资 1758亿元，是“十五”时期的5.2倍，投资对经济增长的贡献率达70%以上。玉柴重机、燕京啤酒、旺旺食品、兴业和北流海螺水泥、容县南方黑芝麻糊扩建等一批重大项目竣工，陆川华润水泥等重大项目加快建设。通江达海交通网络建设取得明显成效，洛湛铁路建成通车，结束了北流、容县不通火车的历史；岑兴高速公路玉林段全面建成，玉林至铁山港高速公路和铁路建设加快推进；全市新增高速公路108公里、一级公路8公里、二级公路358公里。能源、水利、通信等基础设施不断改善。

（五）城镇建设快速推进，城乡面貌明显改观。五年累计完成市政公用设施建设投入104.6亿元，是“十五”时期的8.26倍。市体育中心、江南公园、玉东大道、迎宾大道一二期、人民东路改造、南江桥重建、东门商业广场等一批重大项目建成投入使用。玉林城区建成区面积由48平方公里扩大到56.6平方公里，人口由41万人增加到55万人。全市城镇化率由32.33%提高到38.5%。玉林城区城市绿地率、绿化覆盖率分别由2005年的20.19%、22.75%提高到29.4%和32.26%，荣获“广西园林城市”称号。全面实施城乡清洁工程和城乡风貌改造，城乡面貌焕然一新，实现由“农民城”向区域性现代化中心城市的初步转变。

（六）改革开放全面深化，非公经济蓬勃发展。国有企业、文教卫生、投融资、财税等重点领域改革有效推进；农村税费改革全面完成；农村集体林权制度改革累计核发林权证765.39万亩,完成总任务的73.38%。参与广西北部湾经济区开放开发实现重大突破，龙潭产业园建设取得重大进展，广西银亿科技矿冶公司等一批项目建成投产，再生资源加工利用项目开工建设，林浆纸、能源等一批大型项目前期工作扎实推进。参与西江经济带建设有效推进，创建广西承接产业转移示范市步伐加快。五年共引进项目2268个，合同投资额1350亿元，已到位资金1069亿元。玉林海关建成并开办业务。对外贸易稳步发展，外贸进出口总额由2005年的3.69亿美元提高到2010年的4.51亿美元；五年累计实际利用外资1.7亿美元，是“十五”时期的3.5倍。成功举办第三至第七届玉博会和第一、第二届药博会。创建广西非公经济示范市取得突破，全市非公经济主体由2005年的9万家增加到2010年的13.7万家，从业人员由38万人增加到50万人，非公经济占全市经济总量比重由65%提高到83%。

（七）节能减排有效推进，生态环境日趋优良。万元GDP能耗由2005年的1.51吨标准煤下降到2010年的1.2吨标准煤，年均下降4.51%；五年共削减化学需氧量8.13万吨，削减二氧化硫2.7万吨，全面完成“十一五”污染减排目标任务。市和各县（市、区）污水处理设施全面建成，全市城镇污水集中处理率由2005年的30%提高到80%，城镇生活垃圾无害化处理率由51.11%提高到80%。全市共新建沼气池7.32万座；累计完成植树造林110.61万亩，退耕还林26.4万亩，全市森林覆盖率由2005年的54.2%提高到2010年的59.03%，我市获“广西绿化模范城市”、北流市和容县分获全国、全区“绿化模范县（市）”称号。全市环境质量明显改善，主要河流水质达到Ⅲ类标准，空气环境质量达到国家二级标准。

（八）社会事业全面进步，公共服务不断完善。全市各级财政在社会事业、就业保障等领域的投入累计达231.77亿元，是“十五”时期的2.52倍。深入实施“科教兴玉”和“人才强市”战略。优先发展教育，全面实现城乡免费义务教育，投入资金3.5亿元补助义务教育阶段学校；筹措资金7.22亿元资助家庭经济困难学生105.13万人，实现资助全覆盖；投入资金7.6亿元，完成中小学校舍维修改造和校安工程项目1717个；义务教育毛入学率达到100%；三年职业教育攻坚任务基本完成。实施科技创新计划，建成自治区级以上企业技术中心、工程技术中心10个，市级技术开发中心26个，五年累计获专利授权1564件，比“十五”时期增长122.47%；取得科技成果243项，其中，获国家科技进步奖1项、获广西科技进步奖28项，博白县农科所的感光型杂交稻研究成果获2009年度广西科学技术特别贡献奖；科技特派员工作覆盖全市92%的行政村；科普工作深入开展。人才工作成效显著，建立了2个自治区级、5个市级人才小高地。医疗卫生服务体系建设得到加强，累计投资2.86亿元，完成县、乡、村三级医疗卫生基础设施项目181个，初步解决农村群众就医难问题。累计投入资金7000万元，完成了116个计生服务站所建设，人口计生各项工作实现科学发展，北流市、玉州区获评为全国计生优质服务先进县(市、区)。全市累计完成1313个村点村村通直播卫星覆盖工程，广播电视综合人口覆盖率达93%。建成乡镇综合文化站31个、村级图书室406个、农家书屋400个,文化事业日益繁荣，文化产业发展壮大。群众性文明创建活动深入开展，思想道德建设不断加强。竞技体育和全民健身活动蓬勃发展，建成村级篮球场520个，成功承办了第十一届全国运动会田径预选赛暨2009年全国田径（男子）锦标赛、广西第十一届运动会、广西第十二届少数民族传统体育运动会等全国、全区性重大赛事。《玉林年鉴》获全国第二届年鉴评比一等奖。

（九）民生保障有效加强，人民生活大幅提高。5年新增城镇就业9.2万人、农村劳动力转移就业23.6万人，城镇登记失业率控制在4.3%以内。社会保障水平不断提高，城镇企业离退休人员基本养老金100%按时足额发放；养老、医疗、失业、工伤和生育保险参保人数达136.52万人，比“十五”期末增加77.32万人；新农合参合率92.5%，陆川县2009年被评为全国新农合试点工作先进县。推进新农保试点，试点单位参保率达63.3%。新建乡镇敬老院47个、五保村151个，集中供养达到8000人；累计支付城乡低保金9.23亿元，5.28万城市居民和46.41万农村居民享受最低生活保障，城乡低保实现应保尽保。扶贫开发扎实推进，五年累计减少农村贫困人口9.6万人。革命老区建设取得新成效。新建40个、续建13个水库移民新村，新(改)建移民村屯道路55.88公里。新建农村人饮安全工程703处，解决98.48万人口饮水不安全问题。实施农村危房改造4650户，4211户城镇居民享受廉租住房，5138户城镇居民享受租赁补贴。连续5年开展为民办实事工程，投入财政资金36.2亿元，为民办实事67项，群众最关心的利益问题得到有效解决。战胜历史罕见的冰冻、干旱和洪涝灾害，累计救助受灾群众50多万人次，重建倒塌民房1万多间。城乡居民收入快速增长，城镇居民人均可支配收入由2005年的8297元增加到2010年的17642元，农民人均纯收入由2573元增加到5302元，年均分别增长16.3%和15.6%。物价保持基本稳定。

平安创建取得新成效。认真做好信访和调解工作，“大排查、大接访、大调解、大防控”活动有效开展，社会治安综合治理成效显著，突发公共事件应急处理机制不断健全，全市社会持续和谐稳定。

民主法制建设、国防建设等各项工作取得新成绩。政务、村务、厂务公开深入推进。安全生产形势稳定好转，产品质量和食品药品安全工作不断加强。国防教育和国防后备力量建设深入开展，双拥工作取得良好成效。机构编制、机关事务、新闻出版、国资监管、发展研究、外事侨务、对台事务、民族、宗教、监察、审计、统计、工商、质监、税务、通信、检验检疫、农机、供销、气象、水文、消防、防震、人防、房改、住房公积金管理、档案、修志等各项工作取得新成效，妇女儿童、老龄和残疾人事业健康发展。

政府自身建设迈出新步伐。政府机构改革稳步推进，重大决策程序机制不断完善，政府依法行政水平有新提高。认真执行市人大及其常委会的决议、决定，自觉接受人大、政协和社会监督，共办理人大代表议案、建议520件，政协委员提案999件，办结率100%。市、县、乡镇政务服务中心和村居政务服务站（点）建设加快推进，初步形成了全市政务服务网络。深入开展学习实践科学发展观、创先争优和“工作落实年—绩效攻坚年”等活动。社会管理和公共服务职能进一步加强，电子政务建设取得新成效，“一站式”电子政务新模式获第四届“中国地方政府创新奖”，“中国玉林”政府门户网站获“中国地方网站创新品牌奖”，先后荣获“浙商投资（中国）最佳城市”、“中国粤商最佳投资城市”、“中国创业之城”称号。

各位代表，刚刚过去的2010年，是我市迈向科学发展新阶段的重要一年。我们认真贯彻科学发展观，全面统筹城乡发展，加快转变发展方式，深化改革扩大开放，着力保障和改善民生，全面完成了市三届人大七次会议确定的各项目标任务，经济社会发展迈出了较大步伐。

国民经济保持较快发展。全市生产总值比上年增长15.7%，财政收入增长26.1%，增速均创建市以来新高；全社会固定资产投资增长38.5%，更新改造投资增长87.3%；社会消费品零售总额增长19%；城镇居民人均可支配收入增长11.5%，农民人均纯收入增长17%。

项目推进实现重大突破。玉柴铸造中心一期、开元微型收割机等重大项目相继竣工投产，龙潭至山口一级公路玉林段建成通车，龙潭再生资源加工利用项目、旺仔牛奶等重大项目开工建设。共争取到中央和自治区支持项目1367个，获中央和自治区资金18.83亿元。

城市建设取得较好成效。完成了人民东路延长线、民主北路和大江桥建设改造，秀水路、二环南路建设加快推进，新开工建设文体路、教育东路和清宁北路延长线、玉福大道等道路及狮子山公园、龟山公园三期和湿地公园，进一步提升了城市功能品位。

改革开放合作不断深化。加快融入北部湾经济区和西江经济带开发建设，龙潭产业园新签约项目13个，总投资347.6亿元。全市招商引资共新签合同项目557个，合同金额427.81亿元，增长39.7%。外贸进出口和利用外资上新台阶，全年进出口总额4.51亿美元，增长27.4%。实际利用外资4985万美元，增长3.06%。

社会各项事业全面发展。投入中小学校舍安全工程建设资金1.8亿元，竣工项目358个；落实资金3.11亿元，资助学生30.9万名。建成21个乡镇综合文化站和48个村级公共服务中心、6个国家级乡镇农民体育健身工程，完成103个村点村村通广播电视直播卫星覆盖工程。建成20个县乡计生服务站所，诚信计生工作稳步推进。第三批“整村推进”扶贫开发、库区移民工作取得新成效，50个革命老区基础设施建设项目全部完成。

民生保障得到有效增强。年初确定的十项为民办实事工程基本完成。新农合、新农保覆盖面进一步提高，五大保险参保人数均超额完成自治区下达任务，103个五保村全部竣工。新开工建设廉租住房1313套，新增租赁补贴1428户；农村危房改造开工率达100%。安全生产形势进一步好转。创建社会和谐稳定模范市扎实推进，在全区率先建成市、县两级信访接待中心和乡镇综治信访维稳中心，集中清理执行积案等12项工作获得中央、自治区有关部门表彰。全年没有发生群体性事件，保持了社会和谐稳定。

各位代表，过去的五年，全市人民用勤劳和智慧、用激情与拼搏，谱写了玉林发展的新篇章。五年来，玉林大地发生了翻天覆地的变化，经济社会发展跃上新台阶，各项工作开创了新局面。过去的五年，是我市战胜重重困难、取得新突破、实现大发展的五年，是发展速度最快、效益最好、城乡面貌变化最大的五年，是各项事业不断进步、民生不断改善、人民生活水平不断提高的五年。

回顾五年的奋斗历程，我们深深体会到，实现玉林经济社会又好又快发展，必须坚持贯彻落实科学发展观，全面统筹城乡发展；必须坚持“强柴兴玉”战略，全力做大做强做优工业；必须坚持转变发展方式，全面提升发展质量和效益；必须坚持推进全民创业，加快发展非公经济；必须坚持改革创新，不断增强发展动力和活力；必须坚持扩大开放合作，全面融入北部湾经济区和西江经济带开发建设；必须坚持加强生态文明建设，实现人与自然和谐发展；必须坚持切实保障和改善民生，让人民群众共享改革发展成果。

各位代表，五年的成就来之不易，这是自治区党委、政府和市委正确领导的结果，是市人大及其常委会、市政协监督支持，各民主党派、工商联积极参政议政的结果，是全市干部群众抢抓机遇、团结奋斗的结果，也是历届党委、人大、政府、政协打下的良好基础和市外各方面友好人士支持帮助的结果。在此，我谨代表市人民政府，向全市人民，向中央及自治区直属驻玉单位、驻玉部队、武警官兵、公安干警，向各民主党派、工商联、各人民团体和社会各界人士，向所有关心、支持玉林建设发展的港澳台同胞及海内外朋友们，表示衷心的感谢和崇高的敬意！

“十一五”发展成绩令人鼓舞。但我们也清醒地认识到，我市经济社会发展中还存在不少困难和问题。主要是我市经济总量小，人均水平低，后发展欠发达的基本市情没有根本改变；全市工业化城镇化水平不高，产业结构不合理，基础设施不完善，科技支撑能力不足；发展方式粗放，资源环境压力较大；改善民生和维护社会稳定任务艰巨，政府自身建设有待进一步加强。我们将认真正视存在的问题，在“十二五”时期切实加以克服和改进，把全市各项事业推向前进。

二、“十二五”时期的发展目标和主要任务

“十二五”时期是玉林全面建设小康社会的关键时期，是深化改革开放、加快转变经济发展方式的攻坚时期。我们要树立强烈的忧患意识、机遇意识和追赶意识，凝心聚力，团结拼搏，推动经济社会发展再上新台阶，不断满足人民群众过上更美好生活的新期待。根据市委三届十二次全会通过的《关于制定国民经济和社会发展第十二个五年规划的建议》，市政府组织制定了《玉林市国民经济和社会发展第十二个五年规划纲要（草案）》，提请本次会议审议。

“十二五”时期，全市上下要高举中国特色社会主义伟大旗帜，以邓小平理论和“三个代表”重要思想为指导，深入贯彻落实科学发展观，围绕“富民强市”新跨越，以科学发展为主题，以加快转变经济发展方式为主线，统筹城乡发展，推进工业化、城镇化、农业现代化、信息化、市场化、国际化，深入实施西部大开发战略，积极参与广西北部湾经济区和西江经济带开放开发，推进改革攻坚，扩大开放合作，促进创新创业，保障和改善民生，保持经济长期平稳较快发展和社会和谐稳定，加快建设广西先进制造业基地、现代商贸物流基地、特色农业基地，泛北部湾中小企业名城和创业宜居的区域性中心城市，创建广西非公经济示范市、现代农业示范市和承接产业转移示范市，为实现全面建设小康社会目标奠定坚实基础。

“富民强市”体现了科学发展观的本质要求，与全面建设小康社会的内涵相一致，反映了全市人民的强烈愿望。加快推进“富民强市”新跨越，是我市“十二五”时期的重大任务。《纲要（草案）》提出了推进“富民强市”新跨越的六条基本要求：坚持把壮大经济总量和提升发展质量作为中心任务；坚持把调整优化经济结构作为主攻方向；坚持把加快推进工业化、城镇化发展作为工作重点；坚持把科技进步和科技创新作为重要支撑；坚持把保障和改善民生作为根本出发点和落脚点；坚持把改革开放作为强大动力。

《纲要（草案）》提出“十二五”时期经济社会发展的目标是：

——经济保持平稳较快发展。地区生产总值年均增长10%，力争“十二五”期末实现翻一番；财政收入、全社会固定资产投资、社会消费品零售总额、进出口总额翻一番以上。

——结构调整取得重大进展。工业增加值、第三产业增加值占地区生产总值比重分别提高6.4个和3个百分点，城镇化率提高11.5个百分点，工业化率提高2.1。

——统筹城乡“五年大发展”目标全面实现。“三个集中”取得明显成效，新区建设取得新突破，城乡居民收入、城乡基本公共服务差距显著缩小。

——科技教育发展明显加快。九年义务教育巩固率提高8个百分点，科技进步对经济增长的贡献率提高2个百分点。

——生态文明建设成效显著。单位地区生产总值能耗和二氧化碳排放、主要污染物排放总量削减按自治区下达指标执行。森林蓄积量增加125万立方米，全市森林覆盖率达61%。

——改革开放合作不断深化。重点领域和关键环节改革取得明显进展。非公经济快速发展。参与广西北部湾经济区开放开发和西江经济带、广西桂东承接产业转移示范区建设取得明显成效。

——人民生活全面改善。城镇居民人均可支配收入和农民人均纯收入年均分别增长10％和13％。城镇新增就业14.7万人，城镇登记失业率控制在5%以内。

——社会建设明显加强。社会管理制度、覆盖城乡居民的基本公共服务体系逐步完善，社会更加和谐稳定。

《纲要（草案）》提出了“十二五”规划经济发展、科技教育、资源环境、人民生活4大类共32项具体指标，其中预期性指标16项，约束性指标16项。与以往相比，指标数量减少，重点更加突出，目标更加清晰；将九年义务教育巩固率、耕地保有量、单位工业增加值用水量降低、单位地区生产总值能耗降低、单位地区生产总值二氧化碳排放降低、主要污染物排放减少、森林蓄积量、全市总人口、城镇参加基本养老保险人数、城乡三项医疗参保率等列为约束性指标，体现了科学发展的要求，体现了加快转变经济发展方式的要求，体现了到2020年实现全面建设小康社会目标的要求。

《纲要（草案）》提出了未来五年我市经济社会发展的十二项主要任务：

（一）全面统筹城乡发展，构建城乡一体化发展新格局。大力推进“三个集中”、“六个一体化”，实现统筹城乡“五年大发展”目标，在广西率先基本实现城乡一体化。

加快推进“三个集中”。完善园区基础设施，引导企业向园区集聚发展。逐步把在城镇稳定就业和居住的农民有序转变为城镇居民；开展撤村建居和推进社区建设工程，建设60个新型农村社区，推动60万农民向城镇或中心村集中；积极推进农村劳动力向二三产业转移，到2015年，二三产业就业人员占全部就业人员比重达58%。建立土地流转市场，加强规范管理，引导和推进土地向规模经营集中。

加强城乡基础设施建设。加快完善城乡道路、防洪堤坝、供水供气管网及污水垃圾处理等生态环保设施。抓好郁江引水等项目。实施农村饮水安全工程，到2015年，全市农村自来水普及率达到65％以上。启动新一轮城乡电网改造。推动电信网、广电网、互联网“三网融合”，实施工业化和信息化“两化”融合示范工程，积极发展电子政务。

推进“四大基础工程”。扎实推进农村产权制度改革、农村基层治理机制建设、村级公共服务和社会管理改革、农村土地综合整治。开展农村土地承包经营权登记试点。深化和完成集体林权制度改革。建立7个县（市、区）和玉东新区农村土地流转服务中心、100个乡镇服务站。完善农村金融服务制度，积极发展农业保险。

推进“六个一体化”。全面推进城乡规划建设、产业布局、就业和社会保障、基础设施、社会事业、政策措施等“六个一体化”建设，促进城市基础设施向农村延伸、城市优质资源向农村覆盖、城市现代文明向农村辐射、城乡生产要素自由流动，加快形成城乡经济社会发展一体化新格局。

促进县域协调发展。引导各县（市、区）完善发展定位，加快发展特色产业，争创广西科学发展十佳县，力争进入西部百强县。

积极探索统筹城乡发展体制机制。建立城乡统一的建设用地市场，促进土地增值收益投入向农业农村倾斜。建立新型户籍管理制度和城乡劳动者平等就业制度，完善有利于农村人口转化为城市人口的配套制度和机制。完善农村公共事业长期投入运行机制。

（二）做大做强做优工业，建设广西先进制造业基地。坚持走新型工业化道路，加快发展先进制造业。力争到“十二五”末，工业化率达到4，进入工业化中期。

加快发展千百亿元产业。大力构建“1＋8＋3”工业产业格局。到2015年，力争把机械产业打造成超千亿元产业；把水泥陶瓷、健康、服装皮革、电子信息、矿冶、林产、化工、能源等8大产业培育成超百亿元产业；大力引导发展新材料、新能源、软件与节能环保等3 个战略性新兴产业，力争到2015年，全市战略性新兴产业产值超过100亿元。深入实施“强柴兴玉”战略，力争到2015年，玉柴集团年销售收入超1000亿元、玉柴工业园产值超1000亿元。重点推进玉柴新一代高效环保柴油机和挖掘机及零配件生产、玉州健康产业园、博白旺茂新型建材产业园、广西万象镍铁合金技改扩建、福绵国际服装基地、玉东博华大功率LED路灯等一批重大项目建设。

加快发展临海产业。大力推进龙潭产业园和铁山港东岸码头建设，重点发展再生资源加工利用、有色金属、能源、林浆纸、港口物流等产业，加快推进再生资源加工利用项目、广西银亿镁盐及硫酸项目建设，尽快开工建设林浆纸、重焦沥青及精细化工、华电玉林热电联产等项目，力争到2015年，临海产业总产值达300亿元。

提升园区集聚发展能力。加大园区基础设施投入，重点建设十大园区。加快建设高新技术园区，打造一批特色产业园区。到2015年，工业总产值超1000亿元、300亿元的园区各1个，超100亿元的园区6个，园区产业集中度达到85%左右。

（三）大力发展现代农业，建设广西特色农业基地。依法实施《海峡两岸（广西玉林）农业合作试验区条例》，大力发展生态农业、品牌农业，推动玉林由农业大市向农业强市跨越，加快创建广西现代农业示范市。

加快发展特色优势农业产业。实施“3335”工程，大力发展优质粮食、优质水果、优质畜禽等特色优势产业，建设一批农业产业园区和产业基地，力争到2015年，生猪、三黄鸡、林业等3个产业产值超100亿元，粮食、蔬菜、水果等3个产业产值超50亿元，打造3个10亿元、5个5亿元产业，构建特色鲜明、生态安全的现代农业产业体系。

推进农业产业化经营。重点建设农产品加工园区及基地，加快推进果蔬、中药材和畜禽水产品等一批农产品深加工重大项目，支持南方食品、富英制革、春茂等龙头企业做大做强，到2015年，全市农产品加工率力争达到40%以上，发展5家年销售收入超10亿元、15家年销售收入超5亿元、50家年销售收入超1亿元的龙头企业，农民专业合作社发展到2500个，形成20个特色效益突出的“一村一品”专业村。

加快完善农业基础设施和服务体系。加大水利建设投入，大力推进病险水库除险加固、灌区节水改造配套等农田水利基本建设，加强水土流失综合治理和林业生态建设。抓好农村扶贫开发和革命老区、库区和移民安置区基础设施建设。推进农技推广体系改革和供销社改革，完善动植物保护防疫、农产品检验检测、农产品流通体系和农业信息体系建设。提高农机装备水平。

（四）推动城镇跨越发展，建设区域性现代化中心城市。坚持规划引城、特色立城、产业兴城、文化塑城，全力推动城镇化跨越发展。力争到2015年，玉林城区人口规模达到70万人以上，用地规模80平方公里，全市城镇化率达到50%。

加快城镇规划体系建设。以玉林城区为核心，以县城和重点城镇为节点，加快形成城乡一体化的规划体系。抓好镇村规划，争取用三年左右时间基本实现乡镇、中心村和交通干线沿线50户以上自然村的规划全覆盖。

加快建设三个新区。加快玉东新区、玉柴新区、龙潭新区建设发展。玉东新区重点发展高新技术产业、现代服务业和现代农业，加快完善城市基础设施，建设成为玉林城市新都市、新兴产业的聚集地、统筹城乡发展的示范区、创业宜居的魅力新城。玉柴新区以产业聚集、壮大辐射为重点，建设成为世界级柴油机生产基地、工程机械制造出口基地及国家新型工业化机械生产示范基地，建成工业经济和城市建设同步互动发展的动力新城。龙潭新区以发展临海产业、承接产业转移为重点，推进区城同建，尽快建设成为面向东盟的新型临海产业基地、综合物流保税区和北部湾经济区商贸物流次中心，打造玉林经济发展新一极，带动玉林由内陆城市向临海城市转型，成为玉林产业发展的活力新城。

加快推进“玉-北-福”一体化。根据规划同筹、交通同建、信息同享的要求，以玉东新区、玉柴新区建设为重点，着力推进一批城市主干道、公共建筑、商业设施等建设项目，加快完善“玉-北-福”一体化基础设施。按照开发玉东、优化城西、拓展南北、完善江南、提升城中的思路，拉开城市框架，扩大城市规模，推动新区与旧城协调发展。建设一批城市生态和绿化景观项目，打造城市“绿核”、“绿轴”和南流江、清湾江百里景观长廊，开展创建“国家园林城市”活动。逐步推进“玉-北-福”公交、通信、金融等一体化。到2015年，“玉-北-福”用地规模达150平方公里，城镇人口达160万人，“玉-北-福”一体化中心城市框架基本形成。

加快县城和城镇体系建设。推进各县（市）城区扩大规模，力争到2015年，北流市城区人口达35万人，用地规模34平方公里；博白县城建成区人口35万人左右，用地规模32平方公里；容县县城、陆川县城建成区人口分别达20万人左右，用地规模达19平方公里；兴业县城建成区人口15万人左右，用地规模12平方公里。把北流市、博白县建设成为桂东南次中心城市。加快仁东、成均、民乐、乌石等31个重点镇建设。

加强城镇化管理。深化城乡容貌管理体制改革，建立完善市、区、街道（乡镇）合理分工的城市容貌管理及相应的行政执法体系，深入持久拓展城乡清洁工程，强化公路沿线、乡镇、小街小巷容貌的日常监管及综合整治，不断提高城镇容貌景观整体水平。

（五）加快发展现代服务业，建设广西现代商贸物流基地。《纲要（草案）》提出，要深入实施“商贸兴市”战略，再造“岭南都会”新优势。

提升发展商贸物流业。按照大建市场、建大市场，大建物流、建大物流的要求，规划建设一批大型专业市场、商业街区和物流园区。重点推进玉林豪德现代商贸城、宏进农批市场二期、玉林国际汽车城、富安居家居广场、北流国际陶瓷城、福绵服装城等专业市场建设，改造和完善25家集贸市场，把玉林建设成为南方重要的“菜篮子”和中药材、家装建材、陶瓷、机电、服装饰品的重要交易集散平台，力争到2015年各类专业市场成交总额突破800亿元。重点推进玉林无水港、交通物流基地、机电设备物流中心、瑞安物流园、西药物流配送中心等物流园区建设，力争2015年全市物流业增加值突破50亿元。

加快发展旅游业。重点建设提升大容山国家森林公园、佛子山旅游度假区、都峤山风景区、鹿峰山旅游景区、勾漏洞风景区、福绵梦幻水乡、水月岩-龙珠湖旅游景区、桂东南温泉养生休闲度假旅游带等重点景区，新建广西天河生态园，打造云天文化旅游产业集聚区、玉东都市生态旅游产业集聚区等六大旅游产业集聚区，建设旅游强市。

大力发展会展业和电子商务。重点建设玉林国际会展中心二期工程，完善会展配套设施，加快提升玉博会、药博会，培育发展其他专业会展，形成多种业态、多层次、广覆盖的会展布局。加快发展电子商务。

积极发展各类服务业。大力引进各类金融机构，加快培育发展农商行、村镇银行等具有独立法人资格的地方金融服务机构，积极发展银行、保险、证券、期货、信托等金融业。推动发展法律仲裁、研发设计、营销策划、咨询评估、产权交易等中介服务业。合理发展房地产业。提升发展酒店餐饮业。积极发展社区服务、体育健身、养老保健、教育培训、文化娱乐等新兴服务业。

（六）加快交通基础设施建设，构建通江达海的现代综合运输体系。坚持适度超前原则，统筹发展各种运输方式，加快完善通畅快捷的交通网络。

加快公路网络建设。建成玉林至铁山港高速公路和龙潭至铁山港东岸、二环南路、玉林至陆川、玉林至福绵等一级公路；开工建设荔浦至玉林高速公路、松旺至铁山港东岸高速公路支线，加快推进南宁苏圩至北流清湾高速公路规划建设，畅通出海出省大通道。加快县、乡、村公路建设。到2015年，全市通车公路总里程达9000公里以上，公路网密度每百平方公里70公里以上，其中新增高速公路215公里；实现100%的行政村通硬化路，500人以上的自然村、屯全部通等级公路。

加快推进铁路建设。建成玉林至铁山港铁路和玉林铁路地区扩能改造项目；积极推进贵港至玉林铁路、沙河至山口铁路支线建设，推进实施黎湛铁路电气化和洛湛铁路复线电气化改造，推进张家界经玉林至海口铁路规划建设。到2015年，铁路运营里程达到 455公里，力争开通城际高速列车。

加快推进航道和民用航空建设。重点推进铁山港东岸码头、绣江复航工程规划建设，大幅度提高玉林水运能力。规划建设玉林支线机场。

（七）坚持绿色发展，加强生态文明建设。以节能减排为重点，加强城乡环境保护和生态建设，全面改善城乡人居环境。

加强资源能源节约和管理。到“十二五”期末，全市节能降耗水平和资源综合利用效率达到自治区下达指标要求。完善用水总量控制和定额管理制度。实行严格的耕地保护和节约集约用地制度，坚守全市423万亩的耕地红线。强化林木采伐限额制度，合理开发利用矿产资源。强化节能目标责任考核，在工业、建筑、交通运输、公共机构等领域实施重点节能工程。积极发展水电、风能、太阳能、生物质能等可再生能源。大力发展循环经济，重点在水泥陶瓷、林化等行业构建循环利用产业体系，建设一批循环经济示范企业。

加大环境保护力度。突出抓好污染减排工作，继续淘汰落后产能，提升城镇污水、垃圾处理能力，推进镇村垃圾收运站建设；综合整治工业污染。强化饮用水源地保护，加强地下水污染防治。开展农村环境综合整治。推进重金属污染和机动车尾气污染防治。到2015年，全市空气质量全部达到二级以上，玉林城区和县城集中式饮用水源地水质达标率分别达到100％和90％以上，城镇生活污水集中处理率达85％。

加强生态建设和防灾减灾体系建设。大力推进“绿满八桂”造林绿化工程，加快创建国家森林城市步伐。深入实施生态工业、生态农业、生态服务业、资源保护和利用四大工程，推进山区生态公益林、自然保护区、湿地生态系统建设。推进新一代天气雷达建设，完善地质灾害监测预防和应急救援体系。

（八）深化改革扩大开放合作，提高市场化和国际化水平。全面推进各领域改革，积极扩大开放合作，进一步增强发展活力。

加快重点领域改革。大力推进国有企业和国有资产管理、财税金融、政府采购、资源性产品价格、环保收费等改革，健全要素市场。推进行政体制改革，理顺市与县（市、区）、政府与部门、部门与部门之间权责关系；完善产业园区管理体制。加快城乡社会事业发展体制机制改革，建立完善覆盖城乡的公共服务体系。

全面深化开放合作。以龙潭产业园建设为龙头，突出抓好大产业、大物流等九大领域发展，加快融入广西北部湾经济区。积极参与西江经济带开发，共同推进西江水道、产业、城镇群、生态建设。深化与珠三角合作，加快推进桂东承接产业转移示范区建设。充分发挥海峡两岸农业合作试验区作用，促进玉台合作。拓展与东盟各国的投资合作。推进大招商招大商，吸引国内外大企业大集团来玉投资发展。全面提高对外贸易水平，支持机电、陶瓷等出口产业做大做强做优，积极培育一批亿美元级、千万美元级出口企业，力争到2015年全市外贸进出口总额达9.08亿美元。支持企业“走出去”， 合作开发境外资源。提升利用外资水平，力争 2015年全市实际利用外资达到1.2亿美元。

大力推进全民创业。完善和落实鼓励创业的财政扶持、金融支持等政策措施，大力支持全民创业。深化社会公用事业改革，鼓励引导民间资本进入资源开发、基础设施、公共服务和社会事业领域，拓宽非公经济发展空间。加快建立中小企业信用评价体系，健全融资担保体系，拓宽非公企业融资渠道。进一步用好创业基金，激发全民创业热情，促进微型企业井喷式发展。力争到2015年，全市非公经济主体突破18万户，非公企业达到2万家，非公经济占全市经济总量比重达90％以上，初步建成广西非公经济示范市。

（九）推进科教兴市和人才强市，建设创新型城市。把优先发展教育作为战略基点，加快科技创新和人才开发力度，建设创新型城市。

增强自主创新能力。重点推进机械、水泥陶瓷等千百亿元产业科技支撑工程，14个百十亿元特色农业产业标准化和农产品质量安全工程，水泥、日用陶瓷、矿冶、林化循环经济示范工程等四大科技工程。完善创新体系建设，推进12个国家、自治区级技术中心建设，重点建设玉林国家级内燃机及零部件产品质检中心、国家陶瓷检测重点实验室、玉柴工程技术中心。推进36家科技示范企业建设，积极发展科技型中小企业。全面实施质量兴玉战略，开展重要技术标准研究，大力培育名牌产品。加快完善公共科技服务平台和科技推广服务体系，健全科技投入机制，加强知识产权创造、运用、保护和管理。发展技术市场，促进科技成果转化。大力推进科普活动。完善科技评价激励制度，激发科技人才创新活力。

优先发展教育事业。统筹推进各级各类教育，力争到2015年，学前教育毛入园率达到65%，九年义务教育巩固率达到93%，高中阶段教育毛入学率达到87％，高等教育毛入学率28%左右。促进城乡教育统筹发展，继续推进我市教育发展三年目标，加快推进中小学校建设，优化城区中小学布局，玉林城区五年新建8所中小学校，全市标准化初中达到40%、标准化小学达到50%以上。支持服务好玉林师院建设发展。加强职业教育基础能力建设，规划建设玉林高等职业技术学院和玉柴国际职业技术学院，打造北部湾经济区职业教育基地。加强特殊教育和校外教育工作。大力促进教育公平，义务教育重点向农村、边远地区、贫困山区和薄弱学校倾斜，加大对家庭经济困难学生的资助力度。全面实施素质教育，加强教师队伍建设，提高教育质量。

建设人才强市。加大重点领域、重点区域人才开发创新力度，深入实施玉林学者、特聘专家、人才小高地建设提升等11项人才工程，突出培养造就创新型科技人才，统筹抓好党政、企业经营管理、专业技术、高技能、农村实用、社会工作等各类人才队伍建设，力争到2015年，全市人才队伍总量达到34.6万人。

（十）统筹推进城乡文化建设，构建岭南特色文化强市。大力发展文化事业和文化产业，推进文化精品工程建设，加快构建岭南特色文化强市。

深入开展文明创建活动。深化“和谐建设在基层”活动，加强社会主义核心价值体系建设，突出抓好青少年思想政治教育，提高全市人民的文明素质。积极开展文明城市、文明县城、文明村镇、文明社区、文明单位创建活动，争创自治区文明城市。

促进文化事业大发展大繁荣。规划建设市文化艺术中心、市博物馆、市图书馆新馆、群众艺术馆新馆；完成6个县级文化馆、5个县级图书馆和27个乡镇综合文化站的改扩建工程；每个行政村各建设一个“农家书屋”；基本完成村级公共服务中心建设任务，基本建成公共文化服务体系。落实每个行政村每月放映一场公益电影，每个学期为中小生放映两场爱国主义教育影片。加快公共体育设施建设，推进全民健身和竞技体育发展，到2015年，力争每个行政村都建成一个篮球场，全市体育参与人口达到人口总数的40%以上，国民体质主要指标达到或接近全国平均水平。

大力发展文体产业。扶持发展一批文化企业，重点发展演艺娱乐、印刷出版、教育培训、文物博物、体育健身等产业，推进玉林市广播影视拍摄制作播出基地和杂技艺术培训基地等文化产业基地建设，构建玉林文化产业集中区，推动文化产业发展成为国民经济新的支柱产业。到2015年，力争文化产业增加值占地区生产总值比重达5％以上。

（十一）着力保障和改善民生，大幅提高人民生活水平。《纲要（草案）》提出，要以人民群众最关心、最直接、最现实的利益问题为着力点，努力在保障和改善民生方面取得更大进展。

提高群众收入水平。实施更加积极的就业政策，加大就业岗位开发力度，大力推进自主创业、自谋职业和转移就业，未来五年城镇新增就业14.7万人，再就业1.7万人，农村劳动力转移就业26万人。建立职工收入增长和支付保障机制，积极增加企业退休人员基本养老金，努力提高居民收入在国民收入分配中的比重、劳动报酬在初次分配中的比重。到2015年，力争城镇单位从业人员劳动报酬总额占地区生产总值的比重提高2个百分点左右，月最低工资突破1000元。

提高公共服务水平。健全覆盖城乡居民的社会保障体系，稳步提高保障水平。力争到2015年，城镇企业职工基本养老保险参保人数达33万人，基本医疗保险覆盖率达90%以上；新型农村养老保险覆盖率达50%以上，新农合参合率达90%以上，失业保险参保人数达14.5万人，工伤保险参保人数达15万人，生育保险参保人数达13万人。完善城乡低保制度，合理提高低保标准和补助水平，努力实现应保尽保。深化医药卫生体制和公立医院改革，新增医疗卫生资源重点向农村和城市社区倾斜，建立健全城乡医疗卫生服务体系，提高突发重大公共卫生事件应急处置能力。加强保障性住房建设，未来五年全市建设保障性住房12805套、经济适用住房26.63万平方米、廉租住房17.67万平方米。深化人口与计生综合改革，加强计划生育服务，稳定低生育水平，提高人口素质，遏制出生人口性别比偏高趋势，到2015年，全市人口自然增长率控制在自治区下达指标以内；积极应对人口老龄化。实施新一轮妇女儿童发展纲要，完善残疾人服务体系和保障体系。实施贫困村整村推进扶贫开发。

（十二）加强社会主义民主法制建设，促进社会和谐稳定。坚持党的领导、人民当家作主、依法治国有机统一，扩大社会主义民主，发展社会主义政治文明。

加强民主政治建设。健全民主制度，保障人民的民主权利，扩大公民有序政治参与。认真听取各民主党派、工商联、无党派代表人士的意见，发挥工会、共青团、妇联等人民团体的作用。切实做好民族、宗教和侨务工作。

深入推进依法治市。实施“六五”普法，提高全民法律意识。依法行政，文明执法，加大查办违纪违法案件力度，维护司法公正和社会公平正义。

加强和创新城乡社会管理。引导民间组织有序发展。建设玉林市信访接待中心大楼，优化信访接待环境。强化食品药品和安全生产监督管理，健全突发事件应急体系。健全社会矛盾调解机制，推进城乡社区警务、群防群治等基层基础建设，强化社会治安综合治理，深入开展平安玉林建设活动。

加强国防动员建设和双拥工作。推进军民融合发展，提升国防动员和后备力量建设水平。加强民兵预备役基层建设和人防设施建设，广泛开展拥军优属、拥政爱民活动，再创全国双拥模范城。

三、2011年的主要目标与重点工作

今年是中国共产党成立90周年，也是实施“十二五”规划的第一年。做好今年的工作，对于开好局、起好步，顺利完成“十二五”的各项目标任务意义重大。今年全市经济和社会发展的主要预期目标是：生产总值增长10％，财政收入增长13％以上，全社会固定资产投资增长20％；社会消费品零售总额增长18％，外贸进出口总额增长16％，城镇居民人均可支配收入增长10％，农民人均纯收入增长12％；万元生产总值能耗降低，二氧化碳、二氧化硫、化学需氧量、氨氮和氮氧化物减排按自治区下达指标执行；城镇登记失业率控制在4.3％以内，人口自然增长率控制在9.5‰以内，居民消费价格涨幅5%左右。

实现上述目标，要重点抓好以下十项工作：

（一）大力统筹城乡发展，加快形成城乡互动发展新局面。全力加快新区建设。加快玉东新区13平方公里核心区建设，重点推进玉东大道、人民东路和教育东路延长线、秀水路、文体路等道路和市民广场建设，建成湿地公园，打造创业宜居的综合新城区；修编完善玉柴新区产业规划，推进玉博大道、和强大道、玉发大道等道路以及供水、供电、排污等设施建设，加快产业发展，为推进区城同建打下良好基础；抓好龙潭产业园“三纵五横”主干道及其配套设施建设，重点推进再生资源加工利用园区等重点项目建设，打造玉林经济增长新一极。强力推进“三个集中”。推进工业向园区集中，力争全年新增入园项目180个以上，园区工业总产值达到650亿元以上。完善配套政策，抓好土地综合整治试点和农村社区建设，引导农民向城镇、大村和社区集中居住。积极推进土地向规模经营集中，2011年力争全市土地流转面积累计达到70万亩以上，促进农业规模化发展。积极实施扩权强县强镇。扩大县级部分经济社会管理权限；积极开展扩权强镇试点，赋予扩权镇部分县级经济社会管理权限。鼓励各地根据自身的区位和资源优势，加快发展特色经济。

（二）加快提升现代产业，推动结构优化升级。加快发展先进制造业。鼓励支持企业推进更新改造，力争全年技改投资完成255亿元以上。继续开展园区基础设施建设大会战，加快玉柴工业园、龙潭产业园等重点产业园建设。坚持实施“强柴兴玉”战略，继续支持玉柴加快发展壮大。以玉柴、三环等大企业为带动，大力培育千百亿元产业。力争今年机械产业销售收入突破500亿元，水泥陶瓷、健康产业突破100亿元；力争年产值超50亿元的园区达到3个；年销售收入超10亿元企业达到10家。实施中小企业成长工程，使一大批中小企业销售收入尽快提高到2000万元以上，不断增加全市规模以上工业企业的总量。加快发展现代服务业。加快推进机电设备物流中心建设，开工建设玉林豪德现代商贸城、宏进农批市场二期、玉林无水港、玉林国际汽车城、富安居家居广场等商贸物流园区，改造提升工业品市场等已有专业市场。实施“万村千乡市场工程”，努力扩大城乡消费。积极推进玉博会“四个转型”步伐，着力提升药博会品牌，进一步做大做强会展经济。以北部湾经济区城市旅游联盟、西江旅游联盟为载体，加大旅游宣传推介力度，提升旅游业水平。加快发展文化、信息、科技、金融和中介服务业，大力发展家政服务业。加快发展现代农业。加快推进农业产业发展“3335工程”，发展壮大特色农业产业。抓好12个部级万亩粮食高产示范区建设，实现粮食总产稳定增长。启动“万元增收工程”，促进农民增收。大力扶持农业龙头企业和农民专业合作社发展，力争新增农业产业化经营组织250个以上，总数达到4000个。加强农民职业技能培训，全年力争培训农民达20万人次。深化水利建设体制改革，抓好农田水利等农业和农村基础设施建设，改善农村的生产生活条件。

（三）突出抓好项目工作，力争项目建设实现新突破。全年新开工230个重大项目，确保完成全社会固定资产投资750亿元。突出抓好产业项目。尽快开工建设玉柴新一代高效节能柴油机项目、北流明华皮革制品、博白县尚古陶瓷和新盈邦陶瓷、广西丰沃家具、500KV玉林二变等项目；加快推进玉柴铸造中心二期、龙潭再生资源加工利用项目、兴业海螺水泥二期等项目建设。加快林浆纸等临海产业重大项目前期工作。突出抓好交通项目。加快推进玉林至铁山港高速公路和铁路、龙潭至山口一级公路合浦段等重大项目建设。配合做好南宁苏圩至北流清湾、松旺至铁山港东岸等高速公路和贵港至玉林铁路、黎湛铁路电气化改造的前期工作，力争开工建设荔浦至玉林高速公路。继续开展农村公路建设年活动。加快绣江复航工程、玉林支线机场项目前期工作。突出抓好项目筹资。积极组织申报一批中央投资项目，争取更多的中央投资项目资金。大力引导民间资金投向重大基础设施和重点产业项目。加强与金融机构的沟通对接，加快推进市城投公司发行企业债券工作，不断扩大项目建设融资渠道。

（四）大力推进城镇建设，力求新型城镇化上新台阶。加快推进城乡一体化规划，完成玉东新区一体化规划修编。以玉东新区、玉柴新区建设为重点，大力推进城市基础设施建设，重点加快路网建设，开工建设迎宾大道三期、龟山路、金港大道延长线、江南大道延长线、南流江百里景观长廊、玉柴广场等重大项目，抓好旧城改造，完善城市功能。加快推进各县（市）城区建设，统筹抓好重点镇、中心镇建设，推进新农村建设示范村、特色名镇名村创建活动。深入开展城乡清洁工程和城乡风貌改造，改善城市环境。

（五）全面激活民间资本，加快发展非公经济。完善创业基金扶持政策和管理办法，鼓励支持民间资本投资创业；设立产业发展基金，将符合产业政策、产业关联度大、市场前景好的非公企业技术改造项目纳入全市重点技术改造或重大科技攻关项目，给予技术和资金支持。继续发展小额贷款公司，培育发展和规范融资性担保公司，构建和完善面向非公企业的投融资、科技创新、创业服务等公共服务平台。切实整治投资创业环境，大力发展微型企业，实施中小企业成长工程，促进非公经济快速发展。

（六）大力推进改革开放，增强科学发展动力。深化各个领域的改革。鼓励和推进企业重组，支持玉柴重工、开元、春茂等企业上市。深化金融体制改革，积极开展“引银入玉”工作，大力引进各类金融机构；完善农村金融组织体系和服务体系。全面完成集体林权制度改革。积极稳妥推进居民阶梯电价改革。深化医药卫生体制改革，抓好公立医院改制试点工作，探索组建医疗集团。继续推进教育、文化体制改革，推进事业单位分类改革。积极配合自治区推进玉林市统筹城乡综合配套改革试点、自治区直管县财政体制改革和扩权强县、扩权强镇体制改革。全面融入北部湾经济区开放开发，重点抓好龙潭产业园开发建设，大力培育发展临海产业。积极扩大开放合作，发挥侨乡优势，加强与东盟的对接和合作。主动融入西江经济带开发，突出抓好园区基础设施建设和承接产业转移工作。充分利用玉博会、药博会等平台，加强招商引资，力争实现区外境内到位资金385亿元，实际利用外资突破6000万美元。

（七）积极推进节能减排，加快生态文明建设。加快创建国家森林城市步伐。大力推进造林绿化，提高城市绿化覆盖率。抓好大容山国家森林公园建设。加快建设一批城市生态和绿化景观等重大绿化美化项目。狠抓节能减排不放松。加强国控重点污染源在线监控和重点耗能企业监管，推广先进节能技术和产品，抓好工业、建筑等重点领域节能降耗；进一步整合水泥、陶瓷、造纸等行业，淘汰落后产能。继续建设污水处理管网，有效控制污染物排放；抓好农村畜牧养殖污染治理。加强环境整治和生态建设。加强南流江、北流河、九州江等重点江河以及水源保护区的综合治理，大力推进生态修复工程，加强重点生态功能区保护和管理。加强山洪地质灾害的预警和治理工作，不断完善防灾减灾救灾体系。

（八）大力发展社会事业，促进城乡公共服务均等化。全面实施“科教兴玉”和“人才强市”战略。启动实施玉林市“十二五”科技发展规划，抓好玉林市技术市场、中小企业科技创新孵化服务中心等科技创新公共服务平台建设。大力推进教育发展七项重点工程和教育体制七项改革试点项目，促进各类教育均衡发展。加快玉林高中新校区、玉东新区第一小学等学校建设。全面落实《玉林市中长期人才发展规划纲要（2010－2020）》，抓好各类人才政策的落实，加大重点领域、重点区域和重点工程人才开发力度，加强人才队伍建设，抓紧培养各领域的高层次创新型人才。加快发展文化体育事业。加强思想道德建设，全面深化“和谐建设在基层”活动，培育文明社会风尚。加快推进玉林文化艺术中心、博物馆建设，推进文化市场综合执法体制改革，加快文化事业和文化产业发展。举办“艺术节”、“文化周”、“电影周”等文化活动，活跃基层文化生活。统筹发展其他各项社会事业。抓好广电和信息化工作。加快城乡基层卫生服务体系建设，保障人民群众的身体健康。大力实施人口计生公共服务惠民工程、计生福利工程、优生促进工程和流动人口均等化服务工程，提高全市人口计生工作整体水平。依法保障公民特别是妇女、未成年人和残疾人的合法权益。抓好扶贫、革命老区、库区移民、外事、对台事务、侨务、民族、宗教、统计、人防、气象、地震、水文、史志、档案等工作，促进社会协调发展。

（九）强化民生保障，维护社会和谐稳定。坚持实施为民办实事工程。实行财政向农村和弱势群体倾斜的政策，筹措资金29.01亿元，继续实施十项为民办实事工程。一是实施教育惠民工程。筹措资金3.29亿元，提高九年义务教育巩固率1个百分点，实施校舍安全工程项目150个，对16万名农村义务教育阶段家庭经济困难寄宿生给予生活费补助，更新农村义务教育学校课桌椅40万套，免除就读普通高中的2000名库区移民子女的学费，对3万名中等职业教育学生进行资助，对3000名考上大学的家庭经济困难新生进行路费和短期生活费资助。二是实施文化惠民工程。筹措资金3593万元，建设17个乡镇综合文化站、64个村级公共服务中心、324家农家书屋，在全市行政村每月每村放映一场电影。三是实施医疗卫生和计生保障惠民工程。筹措资金10.72亿元，实施城镇职工和城镇居民基本医疗保险、新型农村合作医疗等城乡三项医疗保险，使参保率达到90%；对参合农民的补助标准提高到年人均200元；为城乡居民免费提供建立居民健康档案、健康教育、预防接种等九类基本公共卫生服务；实施艾滋病防治攻坚和地中海贫血疾病防治工程；支持832个村卫生室标准化建设。安排1200万元资金，建设市计划生育服务中心，推进25个县、乡计生服务站所标准化、规范化建设。支持村级人口计生健康服务室标准化建设。四是实施社会保障惠民工程。筹措资金3.92亿元，进一步扩大城镇企业职工基本养老保险覆盖面，新增参保人员5000人；做好玉州、容县、北流的农村新型养老保险试点工作；提高城乡低保对象的补助标准；新建145个农村五保村。五是实施安居惠民工程。筹措资金6.3亿元，实施城镇保障安居改造4700套；对4650户农村危房进行改造。六是实施生态惠民工程。筹措资金5000万元，重点支持通道绿化和村屯绿化等项目；对31.23万亩国家级和自治区级公益林进行生态效益补偿；补助新建1.4万座沼气池。七是实施强农惠农补贴工程。筹措资金400万元，实施超级稻示范推广项目164万亩；对农民购置农机具继续实施补贴；实施农资综合补贴和农村“一事一议”奖补项目。筹措资金1.65亿元，解决30万人饮水安全困难问题。八是实施强基惠农工程。筹措资金1.46亿元，实施7个土地整理项目建设；支持农业综合开发土地治理项目；推进小型农田水利建设；修建屯级道路342公里，努力解决农民行路难问题。九是实施城乡风貌和城区道路、小街小巷改造工程。筹措资金3000万元，实施12个村村容村貌改造，推进15条城区道路和小街小巷改造。十是实施水库移民新村建设工程。筹措资金3500万元，建设一批水库移民新村。

努力加强社会保障。千方百计扩大就业，力争全年城镇新增就业2.3万人。进一步完善社会保障体系，稳步扩大各项社会保险的覆盖面。全面实施医疗保险市级统筹制度。加快农村危房改造、廉租房和保障性安居工程建设。认真落实国务院16条控价措施，维护正常的市场价格秩序。发展慈善事业，健全社会救助体系。

切实维护社会和谐稳定。组织开展“安全生产基层基础年”活动，加强食品药品安全监管。全面落实属地管理、谁主管谁负责原则和“一岗双责”，深化重点领域专项整治和隐患排查治理；进一步做好信访工作，完善应急管理机制；加强社会治安综合治理，推进平安玉林建设。积极支持部队和国防建设，广泛开展国防教育，深入开展双拥共建，做好优抚安置工作，巩固军政军民团结。

（十）加强政府自身建设，着力提高政府执行力。继续深化创先争优活动，不断巩固“工作落实年－绩效攻坚年”活动成果，进一步提高政府执行力和落实力，推动政府职能向创造良好发展环境、提供优质服务、维护社会公平正义转变。深入整治投资环境。加强政风、行风建设，完善和落实行政审批“一站式”办结、服务企业（项目）直通车等制度，为企业提供“零距离”优质服务。加强政府绩效管理。完善绩效评估制度，健全目标责任体系。推进联合督查，对机关绩效实施全过程精细化动态管理。用好考评结果，强化行政问责，促进工作落实。加强民主法制建设。完善民主决策机制，自觉接受人大及其常委会的工作监督、政协民主监督，认真办理人大代表议案、建议和政协委员提案；重视民主党派、工商联参政议政的作用，充分发挥工会、共青团、妇联等群团组织的桥梁纽带作用。推进法治政府建设，将风险评估结果作为公共决策重要依据，提高依法行政水平。加强廉政建设。切实加强廉洁自律，依法惩治腐败。进一步推进政务公开，加快市县两级公共资源交易中心建设，切实把优质工程、安全工程、阳光工程、廉洁工程的要求落实到工程建设的各个环节，勤勉尽责，努力建设为民、务实、廉洁、高效的政府。

各位代表，“十二五”的宏伟蓝图催人奋进。我们站在新的起点上，肩负新的使命。让我们高举中国特色社会主义伟大旗帜，全面贯彻落实科学发展观，在自治区党委、政府和市委的领导下，团结奋进，锐意开拓，全力拼搏，为实现“富民强市”新跨越而努力奋斗！
